# Enzymatic synthesis of a novel solid–liquid phase change energy storage material based on levulinic acid and 1,4-butanediol

**DOI:** 10.1186/s40643-022-00502-w

**Published:** 2022-02-11

**Authors:** Siyu Zhai, Lihe Zhang, Xi Zhao, Qian Wang, Yin Yan, Cui Li, Xu Zhang

**Affiliations:** Beijing Key Lab of Bioprocess, National Energy R&D Center for Biorefinery, College of Life Science and Technology, Beijing University of Chemical Technology, Beijing, China

**Keywords:** Levulinic acid, Polyol ester, Thermal properties, Enzymatic method, Thermal reliability

## Abstract

The current energy crisis has prompted the development and utilization of renewable energy and energy storage material. In this study, levulinic acid (LA) and 1,4-butanediol (BDO) were used to synthesize a novel levulinic acid 1,4-butanediol ester (LBE) by both enzymatic and chemical methods. The enzymatic method exhibited excellent performance during the synthesis process, and resulted in 87.33% of LBE yield, while the chemical method caused more by-products and higher energy consumption. What’s more, the thermal properties of the obtained LBE as a phase change material (PCM) were evaluated. Differential scanning calorimetry (DSC) and thermogravimetric analysis (TGA) showed that the melting temperature, latent heat of melting, and pyrolysis temperature were 50.51 °C, 156.1 J/g, and 150–160 °C, respectively. Compared with the traditional paraffin, the prepared PCM has a superior phase transition temperature, a higher latent heat of melting, and better thermal stability. The thermal conductivity could be increased to 0.34 W/m/k after adding expanded graphite (EG). In summary, LBE has great potential in the application of energy storage as a low-temperature phase change energy storage material.

**Graphical Abstract:**

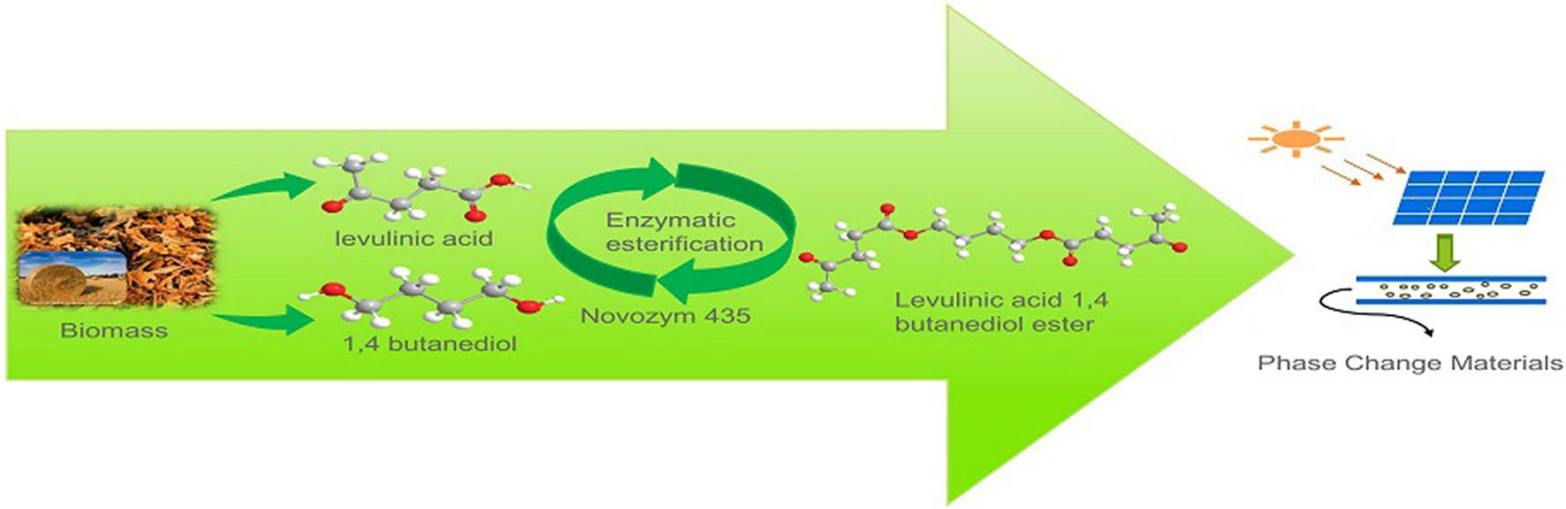

## Introduction

With the rapid consumption of traditional fossil fuels, the Earth received approximately 122,000 TW (70%) of the solar radiation into the upper atmosphere (174,000 TW) (Paul et al. 2021). The energy demand is expected to reach 26.76 TW within the next two decades, so the energy crisis urges people to find more sustainable and clean alternative energy sources (Liang et al. 2021). In recent years, the energy crisis has been alleviated to some extent through the development of solar energy, wind energy, and other renewable energy (Zhang et al. 2018). In the process of the energy transition, renewable energy from the industrial and building sectors has attracted more and more attention (Stamatiou et al. 2017). To better avoid the fluctuation of renewable energy supply, energy storage systems will be an important constituent part of energy consumption in the future (Kant et al. 2016). As a typical energy storage material, phase change materials have received much attention from researchers and industrial enterprises in recent years. Because they can absorb or release a lot of latent heat at their melting point to trigger the increase or decrease of the temperature in the surrounding area (Zhang et al. 2016, 2018; Kant et al. 2016; Wang et al. 2018), Paraffin is a significant thermal energy storage (TES) material (Zhao et al. 2021). It has been used as the basic raw material to synthesize composite phase change materials in the temperature range between low temperature and medium temperature. However, its low thermal conductivity, poor flammability, and non-renewability limit the further application (Sari 2012; Sarı and Karaipekli 2012; Stamatiou et al. 2017; Ravotti et al. 2020). Therefore, most current studies focus on the development of renewable phase change energy storage materials with high latent heat and thermal conductivity.

Levulinic acid, a biobased chemical with carboxyl and carbonyl functional group can be easily produced from glucose, fructose, starch, and lignocellulosic residues (Climent et al. 2014). It has been considered one of the 12 most promising molecules derived from biomass because it could be converted to various important compounds in the chemical industry (Silva 2014; Jeong et al. 2017). The levulinate esters obtained by esterification of LA with some alcohols have a great potential in the application of diesel fuel additive, plasticizer, and flavoring industries (Jones et al. 2016; Dutta et al. 2019; T. Adeleye et al. 2019). However, the esterification between levulinic acid and polyol to prepare phase change materials is rarely reported. In the study, a polyol-BDO, and LA was were used as the raw material to synthesize LBE. Because BDO also can be produced by biological fermentation or biomass conversion (Wu et al. 2017). LBE has an outstanding advantage due to the renewable property of raw materials.

LBE is usually produced by enzymatic or chemical esterification of levulinic acid with polyol (Trombettoni et al. 2017; Zhu et al. 2020). Compared with the chemical method, enzymatic catalysis has many advantages, such as environmental friendliness, less by-products, and mild operation conditions (Mukherjee et al. 2015; Jaiswal and Rathod 2021). However, the reversible reaction and the side product (H_2_O) have an inhibiting effect on the esterification reaction. In order to obtain a high LBE yield, esterification should be carried out in a non-aqueous system (Song et al. 2021). The application of lipase in enzymatic esterification reaction has been developed for many years (Byrne et al. 2021). Enzyme immobilization provides the possibility for recycling biocatalyst, thus reducing the cost of esterification reaction. Moreover, the catalytic activity and stability of the immobilized enzyme were also significantly improved (Moon et al. 2021; Song et al. 2021). The lipase (Novozym 435) was produced from *Candida antarctica*, which is immobilized on a hydrophobic carrier (acrylic resin), the immobilized enzyme shows good performance both in the properties of conversion efficiency and recoverability during esterification and transesterification reactions (Ortiz et al. 2019). For example, butyl butyrate was synthesized using butyric acid and butanol via esterification in a solvent-free system and the yield reached nearly 100% after the optimization of parameters with response surface methodology (Sjöblom et al. 2017). Using hexane as solvent and Novozym 435 as a catalyst, citronellyl palmitate ester was obtained through an esterification reaction with a yield of more than 95% (Ortiz et al. 2019). Enzymatic reactions with organic solvent systems require expensive reactors and tools (Liu et al. 2021), thus limiting the application in the industry (Zhu et al. 2020). The solvent-free system has the advantages of high selectivity and a simple purification procedure. However, using lipase-catalyzed esterification to synthesize LBE in a solvent-free system was rarely studied at present (Jaiswal et al. 2021).

**Fig. 1:**
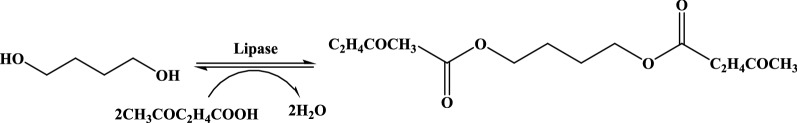
Schematic representation of the esterification reaction between LA and BDO

This study aimed to develop a robust, environmentally friendly, and highly efficient route to produce LBE with renewable materials (LA and BDO), Novozym 435 as the catalyst. The effects of several reaction parameters including reaction temperatures, enzyme dosages, and molar ratios of substrates on the synthesis of LBE and the reusability of the enzyme during the enzymatic reaction process were investigated. The schematic representation of the esterification reaction between LA and BDO is shown in Fig. 1. The LBE was purified by a rotary membrane molecular distillation system. The latent heat properties, thermal stability, and thermal conductivity were measured. The potential of the synthesized esters for solid–liquid phase change energy storage material was also evaluated.

## Materials and methods

### Materials and chemicals

Levulinic acid (99%, wt.%) and 1,4-butanediol were purchased from Adamas Reagent Co., Ltd. (Shanghai, China) and Maclean Biochemical Technology Co., Ltd. (Shanghai, China), respectively. Amberlyst-15 was bought from Aladdin Reagent Co., Ltd. (Shanghai, China), and H_2_SO_4_ (98%, wt.%) was obtained from Beijing Chemical Factory (Beijing, China). Novozym 435 with an initial enzyme activity of 10,000 U/g was provided by Beijing Ruisen Co., Ltd. (Beijing, China). *Candida* sp. 99-125 lipase was provided by Beijing CTA New Century Biotechnology Co, Ltd. (Beijing, China). *Thermomyces lanuginosus* immobilized on silica gel (Lipozyme TLIM) were purchased from Novozym (Beijing, China).

### LBE synthesis

LBE was synthesized by both chemical and enzymatic methods. For the chemical method, H_2_SO_4_ and Amberlyst-15 were used as a homogeneous and heterogeneous catalysts, respectively. The catalysts used for enzymatic synthesis were Novozym 435, *Candida* sp. 99-125, and Lipozyme TLIM. All the reactions were carried out in a three-necked round-bottom flask (250 mL) fitting with a magnetic stirring system at temperatures of 50–90 °C. 0.22 mol LA and 0.1 mol BDO were used as the substrates and the stirring speed was kept at 200 rpm. The reaction conditions were determined according to a previous republication (Ji et al. 2015). The dosages of the catalysts were determined to be 1% H_2_SO_4_, 5% Amberlyst-15, 5% Novozym 435, 5% *Candida* sp. 99-125, and 5% Lipozyme TLIM according to the substrate weight. During the reaction process, dry air was introduced to remove the generated water. Samples were taken out every 1 h to monitor the ester content by gas chromatography (GC).

The catalyst with the best performance was selected for further investigation based on the above results. Then, the effects of esterification time, reaction temperature, molar ratio, and enzyme dosage on enzymatic synthesis were investigated. LA and BDO were mixed in the reactor at a certain mole ratio (1:1.8–2.6), and a certain amount of lipase (1–6%) was added into the reactor subsequently. The ranges of reaction times and temperatures were determined to be 0–8 h and 40–60 °C, respectively. The stirring speed was kept at 200 rpm. Dry air was injected into the reaction system to remove the produced water during the reaction. The ester content was monitored by GC.

After the reaction, the reaction mixture was centrifuged at 8000×*g* for 10 min to obtain the crude LBE product. Lipase was washed for recycling and re-utilization. The crude product was distilled by a Rotary Film Molecular Distillation (VTA GMBH & Co. KG, Germany), with an inlet temperature of 60 °C; a scraper evaporator temperature of 140 °C; central cooling tube temperature of 20 °C; heavy-phase outlet temperature of 30 °C, and 1 bar) to remove the impurities. After that, the obtained product from the first distillation was distilled once again with the parameters of (inlet temperature of 60 °C; scraper evaporator temperature of 200 °C; central cooling tube temperature of 20 °C; heavy-phase outlet temperature of 30 °C, and 0.3 bar) to get the final LBE product with a high purity of 98.31%.

### Analytical procedure

**Fig. 2:**
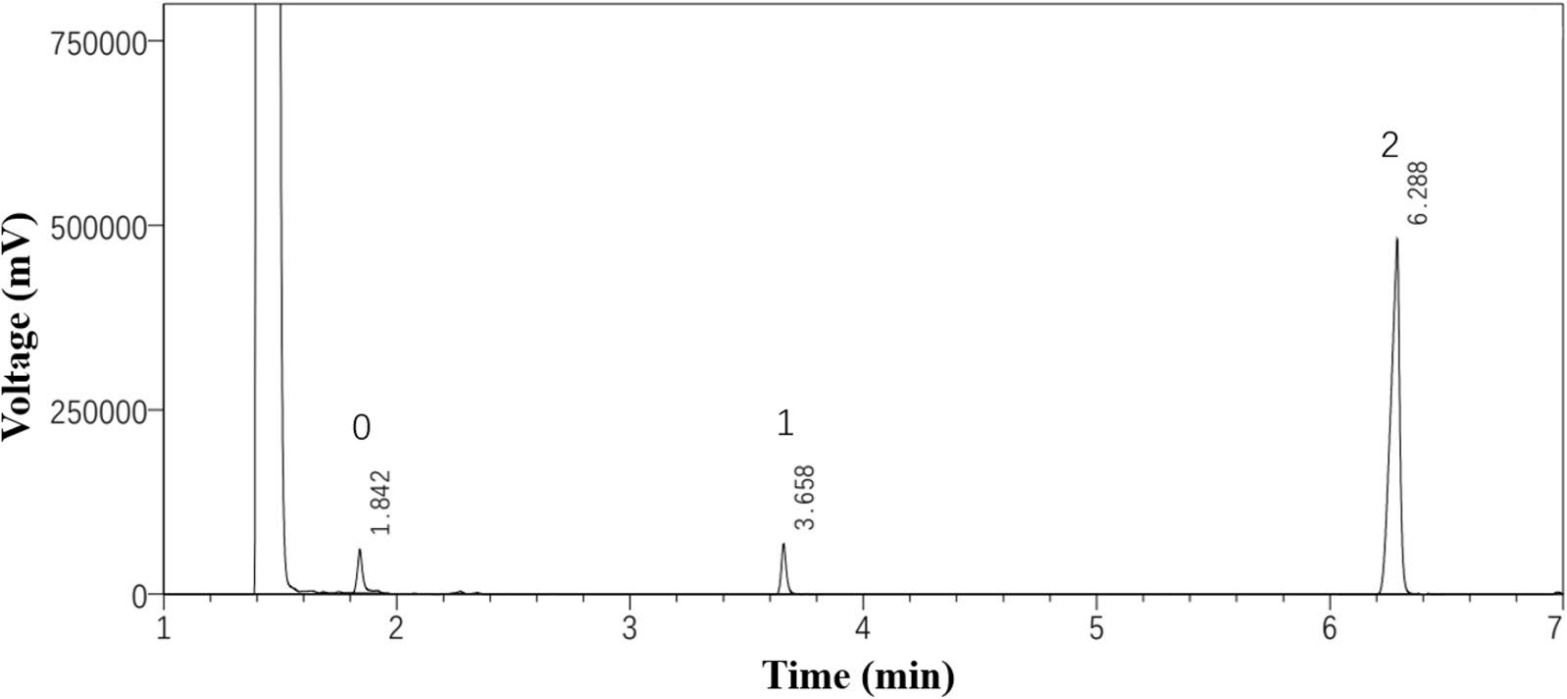
The GC chromatograms of the products during enzymatic esterification. 0—stands for LA (1.842 min); 1—stands for monosubstituted BDO esters (3.658 min); 2—stands for disubstituted BDO esters (6.288 min)

For GC analysis of LBE, the samples were first diluted with ethyl acetate to 1 × 10^4^ ppm and analyzed through Shimadzu GC 2030-FID equipped with a DB-1Ht column (30 m × 0.25 mm × 0.1 µm, Agilent, USA) with nitrogen as the carrier gas, a total flow rate of 53.4 mL/min, and pressure of 138.9 kPa. The split ratio, injection temperature, and FID detector temperature were 30, 300, and 360 °C, respectively. The column temperature was held at 110 °C, then heated to 132 °C at 12 °C/min, and 180 °C at 30 °C/min and finally to 300 °C at 20 °C/min and then maintained for 5 min. The temperatures of the injector and detector were set at 300 °C. The retention time of levulinic acid and mono-, disubstituted BDO esters (Fig. 2) were 1.842 min and 3.658 min, and 6.288 min, respectively. The content of each substance was determined by the area normalization method, and the average value was calculated from three parallel experiments (Zhang et al. 2020).

Nuclear magnetic resonance (NMR) analysis was used for the structural characterization of LBE. First, 0.25 μL of the sample was diluted with 0.5 mL of CDCl_3_ solvent. Then, the diluted sample was transferred into the NMR tube for analysis. ^1^H NMR and ^13^C NMR spectra were recorded using the AV400 NMR spectrometer (Bruker Co., Ltd., GER).

### Thermal performance analysis of LBE

The obtained PCM was characterized by Mettler-Toledo DSC. The sample was placed in an aluminum standard dish for the measurement, and the measured temperature ranged from −40 to 80 °C with a heating rate of 5 °C/min. The DSC analysis is performed under a nitrogen environment with a volume flow rate of 100 mL/min. After the first measurement, the second temperature characteristic curve was tested at the same initial temperature, germination temperature, and heating rate, and the cycle was 30 times to measure the cyclic stability of PCM.

The thermal conductivity of the synthesized PCM was measured by an LFA467 thermal conductivity measuring apparatus at room temperature. TGA was performed on a METTLER thermogravimetric differential thermal analyzer under a flow of nitrogen (flow rate of 40 mL/min) at a constant heating rate of 10 °C/min.

## Results and discussion

### Comparison of chemical and enzymatic synthesis of LBE

Esterification of LA and BDO by the enzymatic and chemical methods was compared under the optimal conditions. The results are shown in Table 1. For the chemical catalysis, the diester contents in the products from sulfuric acid and Amberlyst-15 catalysis were 73.3% and 47.07%, respectively. Although chemical catalysis realized the esterification of LA and BDO, a high reaction temperature of 90 °C was necessary. Moreover, sulfuric acid caused the partial oxidation of raw materials and corrosion of equipment because of its strong oxidizing and acidity (Alegría et al. 2015). Otherwise, the product was difficult to separate from the homogeneous catalyst, sulfuric acid. While using the heterogeneous catalyst of Amberlyst-15 to overcome the difficulty of product separation, the reaction needs to be carried out in a high-temperature environment. The reaction efficiency was as low as 47.07% (Robles-Medina et al. 2009). For the enzymatic method, using Novozym 435, *Candida* sp. 99-125, Lipozyme TLIM as the catalysts, Novozym 435 displayed the highest diester content (85.81%), while the diester contents obtained by *Candida* sp. 99-125 and Lipozyme TLIM were significantly lower (1.32% and 21.91%, respectively). The low affinity of biocatalysts to BDO might be the reason for the low diester content obtained by *Candida* sp. 99-125 and Lipozyme TLIM (Lăcătuş et al. 2018). Novozym 435 is a stable and versatile biocatalyst that has been widely used in esterification and transesterification reactions (Qin et al. 2016). Compared with the chemical method, the enzymatic method presented many advantages such as mild reaction conditions, catalyst recycling, low pollution emissions, and low energy consumption (Badgujar et al. 2016). Therefore, Novozym 435 was selected as a catalyst for further optimization of the reaction.

**Table 1:** Esterification of BDO and LA catalyzed by various catalysts

	Entry	Catalyst	Monoester content (%)	Diester content (%)
Chemical catalyst	1	H_2_SO_4_	7.26 ± 1.23	73.33 ± 0.96
	2	Amberlyst-15	3.26 ± 0.95	47.07 ± 1.21
Biological catalyst	3	Novozym 435	6.92 ± 0.82	85.81 ± 0.81
	4	*Candida* sp. 99-125	12.17 ± 1.25	1.32 ± 0.92
	5	Lipozyme TLIM	5.83 ± 1.31	21.91 ± 1.43

Reaction conditions: 1—0.22 mol LA, 0.1 mol BDO, 1%wt H_2_SO_4_, 90 °C, 3 h, 200 rpm. 2—0.22 mol LA, 0.1 mol BDO, 5%wt Amberlyst-15,90 °C, 7 h, 200 rpm. 3—0.22 mol LA, 0.1 mol BDO, 5%wt Novozym 435, 50 °C, 7 h, 200 rpm. 4—0.22 mol LA, 0.1 mol BDO, 5%wt *Candida* sp.99-125, 50 °C, 7 h, 200 rpm. 5—0.22 mol LA, 0.1 mol BDO, 5%wt Lipozyme TLIM, 50 °C, 7 h, 200 rpm

### Optimization of the reaction conditions

**Fig. 3:**
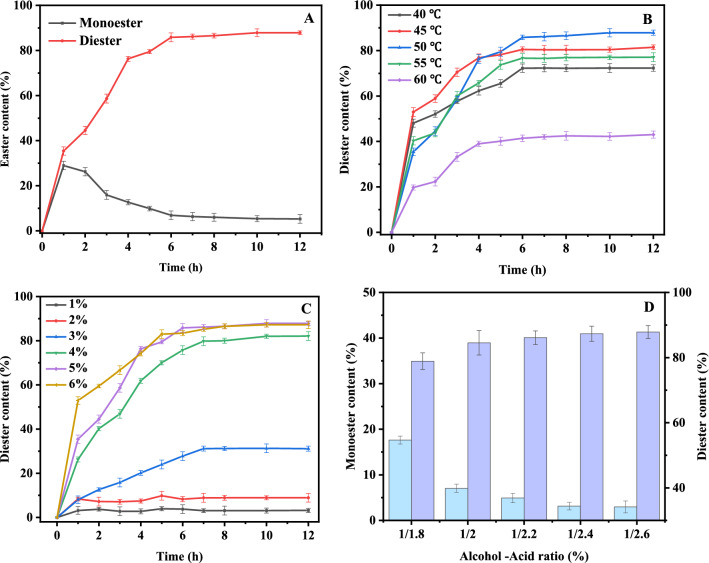
Optimization of reaction conditions for the enzyme-catalyzed synthesis of PCM. **A** under certain conditions, LBE content changes with reaction time; **B** the change of LBE content with reaction time at different temperatures; **C** the change of LBE content with reaction time at different ratios of enzyme addition; **D** the relative percentages of LBE content in the product at different acid–alcohol ratios

In order to obtain a good esterification performance, an enzyme-catalyzed esterification reaction with LA and BDO was carried out under different experimental conditions. Under the reaction temperature of 50 °C, enzyme loading of 5%, alcohol-to-acid ratio of 1:2.2, the effect of different catalytic times 0–12 h on LBE synthesis was analyzed. From the results shown in Fig. 3A, the contents of monoester and diester increased within the first 1 h, the contents of monoester and diester reached 28.94% and 35.43%, respectively. The reason could be concluded that the lipase simultaneously catalyzed the mono and diesterification of LA and BDO. With the increase in diester content, the monoester content gradually decreased during the reaction times of 1–6 h. After 7 h of reaction time, the reaction system tended to reach equilibrium due to the reduction of LA and BDO, and the highest yield of diester reached 86.16%. Therefore, the optimal reaction time for Novozym 435 for the esterification of LA and BDO was 7 h.

In non-aqueous phase enzymatic reactions, temperature affects the enzymatic reaction mainly by affecting enzyme stability, enzyme activity, substrate solubility, and mass transfer resistance. The influence of temperatures (40–60 °C) on the synthesis of LBE was analyzed under the conditions of 5% enzyme load, 1:2.2 of alcohol-to-acid ratio, and reaction for 12 h. According to the results in Fig. 3B, the diester content increased from 72.30 to 87.85% as the temperature increased from 40 to 50 °C. With the increase of temperature, the interfacial mass transfer and the conformational flexibility of the enzyme were improved, and the release rate of water in the system was also increased, which resulted in a high reaction rate. However, when the temperature was raised to 60 °C, the diester content decreased from 87.85 to 43.01%. The reason may be that the high temperature affects the three-dimensional conformation, the intermolecular force of enzyme, and the formation of intermediate complexes, resulting in the reduction of reaction rate and enzyme activity. Therefore, 50 °C was determined to be the optimum reaction temperature for esterification reaction.

The amount of enzyme has a significant impact on diester conversion. Under the conditions of 50 °C of reaction temperature, alcohol-to-acid ratio of 1:2.2, and 12 h of reaction time, the influence of enzyme loading (1–6%) on the synthesis of LBE was analyzed. From the results shown in Fig. 3C, the maximum diester conversion rate was 3.18% with an enzyme loading of 1%. With the increase of enzyme dosage, competitive inhibition was relieved and the reaction rate increased from zero-order reaction to first-order reaction. Therefore, when the enzyme dosage increased from 1 to 4%, the content of the diester increased significantly. When the reaction reaches an equilibrium state, the conversion rate tends to be stable. When the enzyme content increased from 5 to 6%, no significant increase of the conversion rate was found. Therefore, the optimal reaction enzyme dosage is 5%.

The influence of the molar ratio of BDO to LA on the reaction is shown in Fig. 3D. The effect of substrate initial molar ratios (1:1.8–1:2.6) on the synthesis of LBE was analyzed under 50 °C with 5% enzymatic loading for 7 h. The reaction rate significantly increased with a high molar ratio. With the increase of the molar ratio of BDO to LA from 1:1.8 to 1:2.6, the conversion rate of the diesters increased from 78.91 to 87.87%. It was found that the ratio of BDO to LA mainly affected the relative concentration of monoester and diester, which was similar to the results of previous studies (Åkerman et al., 2011). For a certain amount of BDO, increasing LA content led to an increase in LBE yield. When the molar ratio of BDO to LA was 1:2.4, the content of the target product reached 87.33%. No obvious increase of LBE content was found when adding more LA. Therefore, an alcohol–acid ratio of 1:2.4 was identified for the LBE synthesis.

### Recycling of catalysts

**Fig.4:**
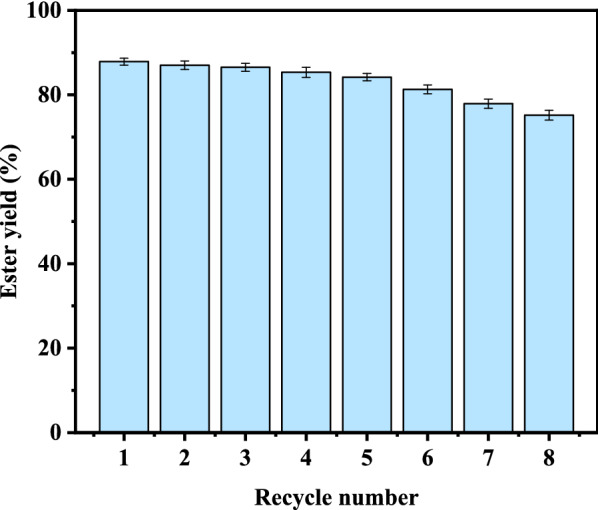
Effects of repeated use of Novozym 435 on LBE yield

Compared with the traditional chemical catalysts, biocatalysts displayed many advantages concluding high selectivity and environmental friendliness. However, relatively high price usually limits their industrial applications. Recyclable immobilized biocatalysts could significantly reduce catalytic costs. Figure 4 shows the reusability of biocatalyst (Novozym 435) during LBE synthesis. When the experiment was repeated for 5 times, the yield of LBE still keep at 84.20% above. After 8 times, the yield of LBE achieved as high as 75.20%. During the experiment, partial damage of the immobilized enzyme particles was observed after 8 repeats, which resulted in a slight decrease of LBE yield. Further development of enzymes with better cyclic stability is needed.

### Structural characterization of LBE

**Fig. 5.**
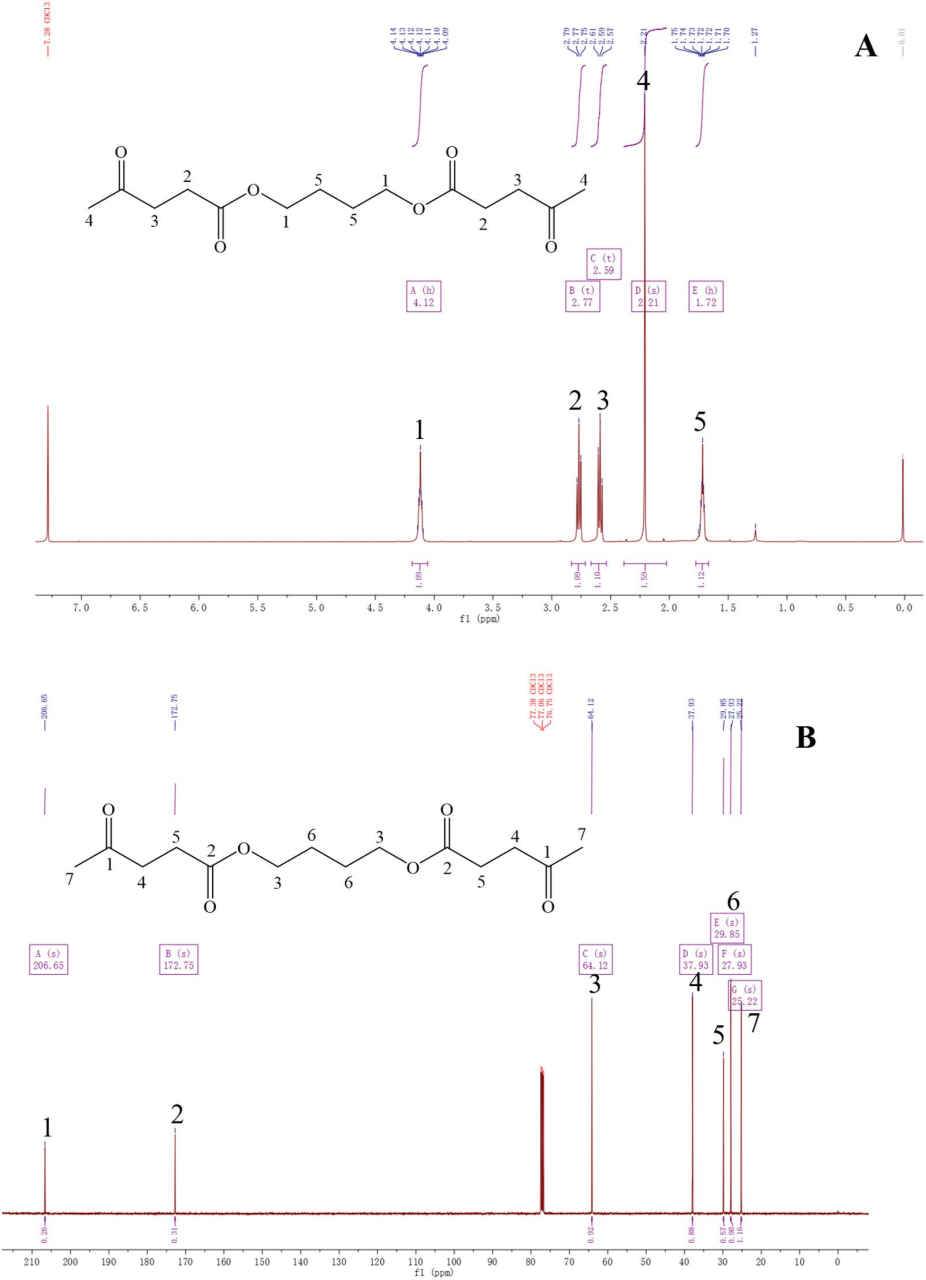
^1^H NMR and ^13^C NMR spectra of LBE (**A** 1; 2; 3; 4; 5 for different hydrogen labeling and chemical structure corresponding to the position. B 1;2;3;4;5;6;7 for different carbon labeling and chemical structure corresponding to the position)

The results of the NMR spectral analysis of LBE are shown in Fig. 5, and the main chemical shifts are given below: ^1^H NMR (400 MHz, CDCl_3_): δ 1.72 (m, 4H), 2.21 (s, 6H), 2.59 (t, *J* = 6.6 Hz, 6H), 2.77 (t, *J* = 6.4 Hz, 4H), 4.12 (m, 4H). ^13^C NMR (100 MHz, CDCl3): δ 206.65, 172.75, 64.12, 37.92, 29.85, 27.93, 25.22. The results of NMR analysis showed that the chemical structure of produced LBE was consistent with its theoretical structure.

### Thermal properties of the prepared PCM

In order to evaluate the performance of LBE as PCM, the melting temperature, thermal melting, thermal conductivity, and thermal stability of LBE were measured and compared with that of a traditional PCM (paraffin).

**Fig. 6:**
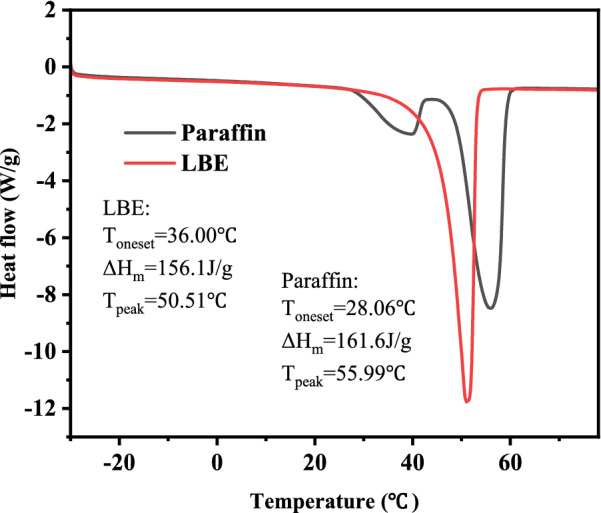
DSC melting curves for LBE and paraffin

The heat storage performance of the synthesized PCM was determined by DSC. Two endothermic peaks of varying sizes were found in the DSC curve of paraffin while only one endothermic peak was observed in that of LBE. From the results shown in Fig. 6, the enthalpy of paraffin melting was 161.6 J/g and the melting temperature was 55.99 °C. The melting enthalpy of LBE was 156.1 J/g and the melting temperature was 50.51 °C. The results showed that the melting temperature and enthalpy of LBE were similar to that of paraffin, which indicated that LBE is a promising alternative in replacing paraffin. And the low transformation temperature of LBE suggests that it can be used as a suitable material for low-temperature thermal energy storage in solar heating applications (Wang et al. 2020). Thermal cycling tests for LBE were also carried out by DSC measurement. After 30 cycles, no obvious changes in the phase transition temperature and latent heat value of phase transition were found. That means the synthesized PCM has a good thermal stability over a long service life.

**Fig. 7:**
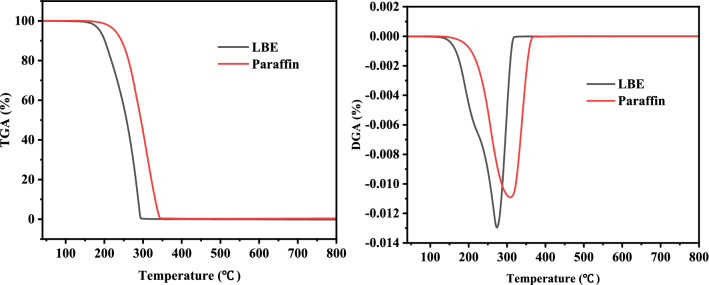
The TGA curves of the LBE and paraffin

The thermal stability of phase change materials is one of the most important parameters in thermal energy storage applications because thermal decomposition, degradation, and sublimation will limit their availability. According to the results in Fig. 7, the thermal stabilities of paraffin and LBE were studied by thermogravimetric analysis. The decomposition temperature of LBE (150–160 °C) was lower than that of paraffin, the latter has been widely used as low-temperature energy storage material. Within the temperature range of below 100 °C, no weight loss and degradation of the phase change material indicated that the PCM has good thermal durability within operating temperature range. Both paraffin and LBE decomposed rapidly at 250–300 °C. When the temperature increased to 300 °C above, LBE was almost completely decomposed without residual residue. Based on the results of TGA, it illustrated that the prepared LBE has good thermal durability for thermal energy storage applications.

The thermal conductivity, phase change temperature, and latent heat values of PCM are also other important parameter in energy storage applications. Low thermal conductivity is the main shortcoming of phase change materials in the heat storage and heat release process. Therefore, low thermal conductivity usually limits the range of PCM applications. Adding graphite or EG into PCM is one of the most effective methods to improve its thermal conductivity because of its high thermal conductivity porous structure, compatibility with organic materials, lightweight (Mazman et al. 2008). The measured thermal conductivities of LBE and paraffin were 0.22 and 0.27 W/m/k at room temperature, respectively. Expanded graphite with high thermal conductivity was added into the prepared PCM with a mass fraction of 5%. The thermal conductivity was improved to 0.34 and 0.31 W/m/k, respectively. The results showed that the thermal conductivities of LBE and paraffin wax were increased by 54.5% and 14.8%, respectively, after adding EG. And the thermal conductivity of LBE is higher than that of the traditional paraffin.

## Conclusions

Thermal energy storage material has attracted more and more attention due to its high energy density and isothermal charge–discharge potential. Therefore, it is essential to develop reliable, cost-effective, and sustainable phase change materials. In this study, a novel polyol ester was synthesized from LA and BDO via enzymatic and chemical methods. The results indicated that the enzymatic method showed a better performance in the synthesis process. The optimal condition of synthesis was determined as follows: reaction temperature of 50 °C, alcohol–acid ratio of 1:2.4, Novozym 435 dosage of 5%, and reaction time of 7 h. Under the optimal condition, the LBE yield reached 87.33%. The thermal properties of the phase change materials were also evaluated. The melting temperature and latent heat of melting of LBE were 50.51 °C and 156.1 J/g. The prepared PCM exhibited a similar transformation temperature and higher melting enthalpy when compared with that of a traditional PCM (paraffin). All samples decomposed above 150–160 °C and had good thermal stability under low temperatures. At room temperature, the thermal conductivity of the paraffin and the prepared PCM were 0.22 and 0.27 W/m/k, respectively. With the addition of EG, the thermal conductivity of LBE was higher than that of paraffin wax, reaching 0.34 W/m/k. Unlike inorganic PCM, LBE has no phase separation and no corrosion. In addition, LBE has completely consistent phase transition behavior and good cyclic stability. The biologically based raw materials and the mild synthetic process conditions make it a more sustainable option than other types of PCM.

## Data Availability

All data are fully available without restriction.

## Abbreviations

BDO: 1,4-Butanediol
LA: Levulinic acid
LBE: Levulinic acid 1,4-butanediol ester
PCM: Phase change material
DSC: Differential scanning calorimetry
TGA: Thermogravimetric analysis
TES: Thermal energy storage
Novozym 435: Immobilized *Candida Antarctica* lipase B
EG: Expanded graphite
Lipozyme TLIM: *Thermomyces lanuginosus* immobilized on silica gel
